# A qualitative examination of substance use service needs among people who use drugs (PWUD) with treatment and service experience in Ontario, Canada

**DOI:** 10.1186/s12889-021-12104-w

**Published:** 2021-11-06

**Authors:** Cayley Russell, Farihah Ali, Frishta Nafeh, Sean LeBlanc, Sameer Imtiaz, Tara Elton-Marshall, Jürgen Rehm

**Affiliations:** 1 Institute for Mental Health Policy Research, Centre for Addiction and Mental Health (CAMH), #2035-33 Ursula Franklin St, Toronto, Ontario M5S 2S1 Canada; 2 Drug Users Advocacy League (DUAL), 216 Murray St, Ottawa, Ontario, K1N 5S6 Canada; 3 Dalla Lana School of Public Health, University of Toronto, 155 College St, Toronto, Ontario M5T 3M7 Canada; 4 Department of Epidemiology and Biostatistics, Schulich School of Medicine and Dentistry, Western University, 1151 Richmond St, London, Ontario M6A 5C1 Canada; 5 Campbell Family Mental Health Research Institute, Centre for Addiction and Mental Health (CAMH), 250 College St, Toronto, Ontario M5T 1R8 Canada; 6 Department of Health Sciences, Lakehead University, 955 Oliver Road, Thunder Bay, Ontario P7B 5E1 Canada; 7 Department of Psychiatry, University of Toronto, 1 King’s College Circle, Toronto, Ontario M5S 1A8 Canada; 8 Institute of Medical Science (IMS), University of Toronto, 1 King’s College Circle, Toronto, Ontario M5S 1A8 Canada; 9 Institut für Klinische Psychologie und Psychotherapie, Technische Universität Dresden, Chemnitzer Str. 46, 01187 Dresden, Germany; 10 Department of International Health Projects, Institute for Leadership and Health Management, I.M. Sechenov First Moscow State Medical University, Bol’shaya Pirogovskaya Ulitsa, 19с1, Moscow, Russia 119146

**Keywords:** Addiction, Harm reduction, Needs, Ontario, People who use drugs, Policy, Service provision, Substance use, Treatment

## Abstract

**Background:**

People who use drugs (PWUD) often have complex health and social support needs related to substance use, yet face numerous barriers to service access, resulting in unmet treatment needs and a corresponding gap in treatment. While initiatives to scale up substance use services for PWUD in Canada - and Ontario - have been undertaken, these have excluded PWUD’ perspectives, and their needs have largely been defined by other actors. As end-users of services, PWUD’ perspectives are vital to understanding what services are required, and whether existent services are adequate, appropriate and effective. Thus, the present study aimed to elicit in-depth knowledge from PWUD with lived experience of accessing services to better understand their unmet treatment and service needs, towards closing the service and treatment gap in Ontario.

**Methods:**

This qualitative study included one-on-one interviews conducted with a cohort of *n* = 45 adult PWUD with substance use and treatment experience in Ontario, Canada. Participants were recruited from substance use services based on ConnexOntario’s directory of all provincial addiction services, as well as by word-of-mouth. Questions focused on participants’ experiences and perspectives on substance use services towards understanding their service needs. Data underwent an inductive thematic analysis based on key themes that emerged.

**Results:**

Participants commonly engaged in polysubstance use, and identified a number of unmet substance use service needs including complex factors within the current service system that influenced access to available programs. Specifically, participants suggested the need to address stigmatization and system fragmentation, increase service provision and capacity, and scale up specific services and related supports such as harm reduction, counseling, treatment, and housing.

**Conclusions:**

This study identified PWUD’ needs in relation to substance use service provision in Ontario, Canada, and highlighted important areas for policy change and program planning and implementation. Concrete recommendations include the development of a government-funded, low-barrier, comprehensive and integrated service delivery and referral models that include PWUD as collaborators and program facilitators to ensure that services are as accessible, effective, and cohesive as possible. Results from this study can be used to enhance provincial substance use treatment and service provision.

**Supplementary Information:**

The online version contains supplementary material available at 10.1186/s12889-021-12104-w.

## Background

People who use drugs (PWUD) often have an elevated prevalence of substance use disorders and co-occurring chronic conditions such as mental health issues (e.g., mood disorders) and infectious diseases (e.g., HIV, Hepatitis) [1–7]. As such, PWUD commonly have complex health care and social support needs related to substance use, which are often complicated by intersecting structural vulnerabilities (e.g., poverty, homelessness) [8–10]. For instance, substance use can create competing survival needs among PWUD when resources are limited [11], and residential transience has been found to shape vulnerabilities related to substance use outcomes (e.g., injection drug use) [12]. Thus, PWUD require access to comprehensive health care, social support, and substance use-related services in order to improve their health and well-being, yet research highlights that PWUD consistently sustain unmet treatment needs [13]. For instance, a global review found that the vast majority of people affected by substance use disorders do not receive treatment, and that this gap remains among the most extensive of all disorders [14]; Further international literature confirms that the unmet need for mental health and substance use care is universally high [15]. Additionally, a US-based population study reported that among 7.7 million US adults with co-occurring mental health and substance use disorders, nearly 90% did not receive substance use treatment in the past year, indicating a vast unmet treatment need among this vulnerable population [13]. As a main contributor to unmet treatment needs, PWUD experience numerous barriers to accessing substance use treatment and supports. For example, research has identified the problematic effect of drug prohibition on PWUD’ willingness to seek support [16–19], as well as socioeconomic, psychological, and structural barriers, such as stigma, affordability, wait times, hours of operation, and service availability, as key barriers to treatment access [20–25]. The combination of unmet needs and barriers to treatment access have resulted in a critical treatment gap; the narrowing of which is essential to ensure that PWUD receive timely and effective care.

While the treatment gap remains prevalent globally, it is particularly pronounced in the Canadian context. For example, available population data indicate that two-thirds of Canadians with a substance use disorder did not seek any care for their substance use or related issues in 2012, commonly due to service models that fail to provide coordinated care for PWUD’ complex needs [26–28]. In recent years, substance use and related harms (e.g., opioid-related hospitalizations, overdoses, and mortality) have escalated substantially across the country, yet rates vary across the provinces [29]; concretely, the province of Ontario reported the highest number of opioid-related overdose deaths nationally in 2019 and 2020 [30]. These realities underscore the potential for an amplification of unmet service and treatment needs among PWUD in Ontario [30, 31]. Indeed, according to the most recent data available from Ontario’s Drug and Alcohol Treatment Information System, the demand for substance use treatment has been increasing across the province, where approximately 65,000 PWUD (only 1 in 218 Ontarians) accessed publicly-funded treatment in fiscal year 2017/18 [32]. Although PWUD in Ontario come from diverse socio-economic, demographic, and cultural backgrounds, the majority of those who seek treatment are young (median age of 34) men (62%) who present with alcohol-related problems (~ 70%) [32], while older, women, gender-diverse, and PWUD with polysubstance use are commonly underrepresented, resulting in a lack of data on these particular PWUD populations [33].

In response to the rise in opioid-related harms and demand for treatment, the Canadian federal government recently undertook a number of initiatives. These included amending the federal drug strategy to increase access to harm reduction services, expanding public awareness campaigns to reduce the devastating impact that stigmatization has on PWUD, and providing emergency funds to the provinces to improve access to evidence-based treatments [34–37]. However, significant variations in substance use policy and program delivery across the country persist, in part due to Canada’s decentralized health care system, where provinces and territories are primarily responsible for the funding, planning, and delivery of health services, including substance use-related services and treatments for PWUD [38–40]. Specifically, in Ontario, the Ministry of Health and Long-Term Care is accountable for funding publicly available substance-use related services (e.g., addiction treatment, harm reduction, rehabilitation programs, etc.), which are delivered through Local Health Integration Networks and community organizations [41, 42]. These public substance use services are financially covered at the individual level by Ontario Health Insurance Plan (OHIP); however, wait times can be long and spaces are often limited [43]. Additionally, privately-funded residential treatment centers exist, but these must be paid for out-of-pocket by individuals, or through employment insurance (where possible), rendering these services largely inaccessible to a large proportion of PWUD [44]. Despite the availability of both public and privately-funded substance use services in Ontario and the increase in treatment demand, stigma and criticism continue to contribute to a lack of public and political support for the expansion of evidence-based substance use services (e.g., harm reduction) [37, 39, 45, 46]. This lack of support has hindered broad implementation of evidence-based policies and programs in the province, and these factors suggest that PWUD in Ontario may continue to experience a gap in treatment.

Importantly, policy and programming initiatives and decisions aimed at closing the treatment gap in Canada and Ontario have systematically excluded PWUD’ perspectives, and their needs have largely been defined by other actors. However, current literature highlights that research and service implementation which engages PWUD throughout program development and delivery processes can more effectively address their needs, and result in beneficial public health outcomes such as reduced relapse rates, increased treatment retention, improved relationships with treatment providers and social supports, and increased satisfaction with treatment experiences [47–53]. For instance, a review examining the effectiveness of engaging PWUD in policy and program development found that services and interventions run by PWUD are not only able to reach more diverse and underserved PWUD populations, but they also decrease risk behaviors among injection drug users [50]. Therefore, as end-users of services, PWUD are crucial actors in identifying potential issues in service and treatment provision, as well as appropriate responses that will meet their specific needs [53].

In light of the increasing harms related to the opioid overdose epidemic, as well as recent policy changes and the widening treatment gap in Ontario, it is important to gain an understanding of the current substance use service needs among PWUD who utilize addiction treatments and services in the province. Thus, the present qualitative study aimed to elicit in-depth knowledge from PWUD who have accessed these services in Ontario in order to enhance our comprehension of their unmet treatment and service needs. This information can be used to inform the evidence base for optimal substance use care provision and planning, and can support policies aimed at closing the service and treatment gap in Ontario.

## Methods

### Design and assessment tools

The current qualitative study utilized a semi-structured one-on-one interview guide (see supplemental material) to elicit information on participant experiences and perspectives on substance use services in Ontario, including specifically related to service needs. Questions examined services PWUD had utilized, how they were connected with services, what services they found beneficial and why, any challenges/barriers they encountered while accessing services, as well as suggestions towards improving service provision as based on service and treatment needs.

### Recruitment

In order to recruit participants, we compiled a list of all available addiction-related services in Ontario based on the database maintained by ConnexOntario, a province-wide information and referral service for substance use services. The final list included *n* = 235 adult-based substance use related services and organizations in Ontario. A member of the research team called all services to identify a key contact who would be willing to assist with recruitment activities. We were able to establish a key contact at *n* = 64 of the organizations (representing a 27% response rate), who were sent an email containing further details about the purpose of the study and a copy of the digital recruitment material (i.e., study poster). Key contacts varied in occupational role at each organization, but were required to be a public-facing member of the organization who dealt directly with clients. Key contacts supported our recruitment efforts by posting and distributing the recruitment materials onsite at their service location and/or on social media platforms, as well as by providing study details directly to potential participants via word-of-mouth. Interested PWUD then either called or emailed the study line/email address where they were screened for eligibility, and an interview was scheduled with eligible participants.

The final breakdown of services from which participants were recruited from included: inpatient/residential treatment programs (*n* = 18); outpatient/aftercare treatment programs (*n* = 13); peers/social media (*n* = 8); opioid agonist treatment (OAT) clinics (*n* = 2); drop-in community addiction clinic (*n* = 1); self-help group (*n* = 1); withdrawal management (*n* = 1); and a shelter (*n* = 1).

### Eligibility and consent

Study eligibility criteria included individuals who were: 1) adults aged 18+; 2) residents of Ontario; 3) fluent in English; and 4) past and/or current users of illicit and/or non-medical drugs (including opioids, stimulants, hallucinogens, benzodiazepines, and/or polysubstance users) and 5) past and/or current users of substance use treatments or services in Ontario. Individuals were excluded if they solely used licit substances (i.e., alcohol and/or cannabis) as we were interested in examining service needs of illicit and high-frequency/polysubstance users.

Prior to commencing the interview, study staff explained the study details in depth to each participant, allowed for questions to be asked, and acquired informed verbal consent (for interviews conducted over the phone) or written consent (for interviews conducted in person) from each participant.

### Participatory action research approach

In order to ensure our study and the results were grounded in the experiences of the members of the communities affected by our research, and whose expert voices should be the primary driver behind any policy and/or program design decisions, we conducted this study in meaningful collaboration with PWUD. Through longstanding connections with cohorts of Ontario-based PWUD that were developed and fostered by the Ontario node of the Canadian Research Initiative in Substance Misuse (CRISM), the research team was able to assemble a project steering committee of PWUD who assisted in the development and design of the study, the interview guide, the interpretation of results, and the final manuscript. The PWUD steering committee worked with the research team iteratively on each project item on an ongoing and collaborative basis, providing informative feedback and suggestions throughout the entirety of the research project. This is in line with current best practices when conducting community-based participatory research and valuing the inclusion of PWUD in research [51, 54, 55].

### Data collection

Between October 2019 and February 2020, a member of the research team (CR) trained in qualitative interviewing techniques conducted all interviews either in person in a private interview room (*n* = 18) or over the phone (*n* = 27). Each interview took approximately 30 min to 1 h in length. Taking into consideration the appropriateness of compensation for this particular group, and administrative requirements (i.e., participants residing within residential treatment centres were not allowed to receive cash), participants received a $30.00 gift card honorarium to a service of their choice as compensation for their time, effort, and involvement in the study.

### Data processing and analysis

The study was confidential and all personal information were de-identified. Participants were provided with a unique code and all personal identifiers were kept separately and removed from the data. All interviews were audio-recorded and subsequently transcribed verbatim and imported into qualitative data management software (NVivo, version 12). Interview transcripts underwent an inductive thematic analysis whereby key themes were identified and subsequently coded. Initial themes were developed based on our research questions, and a coding scheme and codebook was created. All interview transcripts were independently reviewed and coded by a single member of the research team (CR). In order to ensure inter-coder reliability, an independent coder (FN) coded a randomly selected sample of the transcripts. Any coding discrepancies were discussed and agreed upon until our data met criteria for qualitative data saturation. To assure the results were adequately represented, interpreted, and transparent, an iterative feedback process was undertaken and all preliminary themes were reviewed by our PWUD advisory group. Select illustrative quotes are provided to narratively represent the major themes which arose.

### Ethics approval and consent to participate

The study protocol and all procedures were approved by the Centre for Addiction and Mental Health (CAMH) Research Ethics Board (REB# 064/2019). Written informed consent was obtained from all subjects and all methods were performed in accordance with the relevant guidelines and regulations.

## Results

### Sample

A total of *n* = 45 PWUD across Ontario participated in the study, ranging in age from 22 to 63, with a mean age of 37 years (See Table 1). The majority (58%) were men. Most (71%) of the participants indicated polysubstance use (i.e., use of more than one substance).

**Table 1:** Sociodemographic Characteristics and Substances Used among Participants (*n* = 45)

Characteristic	***n*** (%)
**Gender**	
Men	26 (58%)
Women	19 (42%)
**Mean age**	37
Substances used*	
Polysubstance use	32 (71%)
Cocaine	27 (60%)
Crack-cocaine	20 (44%)
Opioids	20 (44%)
Cannabis	12 (27%)
Alcohol	11 (24%)
Methamphetamine(s)	7 (16%)
Hallucinogens/Party Drugs	4 (9%)
Benzodiazepines/Sleeping Pills	4 (9%)

*Substances used were not mutually exclusive. Percentages are based on qualitative responses to participants’ ‘most commonly used substance(s)’. Opioids included synthetic and non-synthetic (heroin, morphine) opioids. Methamphetamine(s) included crystal meth and speed. Hallucinogens/party drugs included MDMA, LSD, GHB, Inhalants, and psilocybin. Benzodiazepines included reference to ‘benzos’ as well as specific brand names such as Xanax, or sleeping pills more broadly

In terms of regional distribution of participants, these have been categorized based on Ontario’s new interim transitional Local Health Integration Network (LHIN) regions (see Table 2) [56].

**Table 2:** Ontario Regional Distribution of Participants based on LHIN Regions (*N* = 45)

Interim Local Health Integration Network (LHIN)	Participant n (%)
North LHIN	13 (29%)
East LHIN	10 (22%)
Central LHIN	16 (36%)
West LHIN	3 (7%)
Toronto LHIN	3 (7%)

### Qualitative results

All participants either had a history of engagement or were currently engaged in services for illicit/non-medical drug use. Level of engagement and services used varied across participants based on demographic factors as well as personal experiences, trajectories, and goals in regards to substance use. When examining services ‘needed’, participants provided concrete suggestions and recommendations towards scaling up and implementing necessary substance use services and supports. These needs have been categorized below under the following subheadings: *Address Stigmatization; Address System Fragmentation; Increase Service Provision and Capacity; and Scale-Up Specific Services and Supports such as Harm Reduction Services, Counseling, Treatment, and Housing.*

#### Address stigmatization

One of the key issues participants discussed was the ways in which stigmatization affected whether or not they would seek treatment, and whether they found support beneficial or not. Many participants discussed experiencing different forms of stigmatization – including social stigmatization, structural stigmatization, and self-stigmatization – for their substance use:

*“There is a shame attached to the stigma of being an addict and asking for help.” (Participant 04; 44-year-old man)*

Participants often indicated that they had experienced structural stigmatization via judgmental staff members at different service organizations, and provided a variety of negative experiences, many of which discouraged them from seeking further help. Others discussed social stigma and reported being embarrassed or ashamed to seek help, explaining how no one in their community was willing to openly discuss substance use and related issues. This lack of open discussion contributed to feelings of internalized stigma, which in turn led participants to hide their substance use from their friends and families, and in some cases, use in riskier ways.

As such, participants discussed a distinct need to address and ‘break down’ stigma. Suggestions included ensuring services employed empathetic and caring staff who offered non-judgmental support, as well as conducting more research into understanding how stigma can affect people. Additionally, disseminating knowledge on the complexities of substance use, and how stigma may perpetuate PWUD’ unmet service needs were suggested, including specifically educating medical and front-line professionals who work with PWUD on this topic:

*“I feel like especially [among] medical professionals, there’s a huge stigma and neon sign put on your forehead if you say you’re an addict. You’re treated a certain way and it’s not okay. If you’re in there and you want help, I think that there shouldn’t be judgement. There’s a lot of information about addiction that’s not in their curriculum, it’s just not something that they learned, so there’s a judgment placed.” (Participant 38; 30-year-old woman)*

#### Address system fragmentation

Beyond stigmatization, participants commonly detailed accounts of major gaps in service delivery, including a glaring lack of service coordination, and discussed the general fragmented nature of Ontario’s substance use service system. Many participants indicated that transitions between services were not seamless, which was especially problematic. Examples included a lack of referral or being referred to the wrong or unhelpful service, a lack of follow-up, and having to go to multiple different services to find assistance:

*“Especially when it comes to opiates, they need everything to happen seamlessly. It’s just one referral system to another and people get lost in the system.” (Participant 12; 33-year-old woman)*

This issue was particularly apparent for the linkage between detoxification and residential treatment. Participants explained that once admitted into detox, they could only stay a limited amount of time, after which they were expected to transition into residential treatment. However, capacity issues often precluded them from direct admittance into residential treatment. This resulted in participants being released from detox and often returning to substance-conducive living situations, which led to feelings of discouragement, fear, and frustration. On many occasions, they would relapse and have to start the entire process over again:

*“I had gotten into a couple detoxes, and then I was trying to get into rehabs, but it made no sense because once I went to detox I had to wait months sometimes to get into [treatment].” (Participant 13; 35-year-old man)*

Other illustrations included limitations on the number of days allowed at certain services, ageing-out of services (i.e., young adults being forced to transition out of treatment due to their age), or having to undergo a period of detoxification before being allowed to enter treatment, which often left participants in a treatment void and were considered major hurdles to accessing support. As such, many participants felt there was a need to address what they considered was a ‘broken’ system, and the common experience of 'falling through the cracks' of the service system. Suggestions to remedy this issue included improving service coordination by enabling seamless transitions between services. As an example, one participant suggested:

*“I think, ideally, I guess just to make it seamless. It’s almost like you need something all in one. I had a friend who overdosed and died 6 months ago. He had to get to the detox, they sent him to the hospital … he had problems getting into detox and had to wait a week. So the whole process isn’t seamless. The system kind of fell through for him. There’s a fundamental hole in the system. It’s just one referral system to another and people get lost in the system.” (Participant 11; 37-year-old man)*

#### Increase service provision and capacity

Other common needs that were conveyed related to aspirations for services to increase their ability and capacity to serve more people at a time, in a less administratively onerous and burdensome way. For instance, many participants described the need for detoxification services/withdrawal management to increase the number of beds available, and reduce the amount of time and energy required for admittance:

*“They say call every hour [to get into detox]. I normally call every 30 minutes. Like I understand that there’s situations that other people are already there, but a lot of people use them as shelters too, right?” (Participant 28; 28-year-old man)*

Additionally, capacity issues were common at detox and shelter locations, where many participants would use these services interchangeably when they could not secure a place to sleep. However, participants reported they often had difficulties being admitted to detox facilities due to stringent admission requirements, such as being acutely intoxicated:

*“[The detox] was literally a place to sleep. The shelters were full and the detox wouldn’t take you unless you’re intoxicated, so I made sure I was intoxicated so I could stay somewhere overnight. When the shelters get too full, it’s kind of scary. I literally got turned down from a detox because I was sober. So I said here, wait a couple hours, I’ll be back.” (Participant 35; 33-year-old man)*

Additionally, participants described numerous accounts of having to wait to receive support at virtually every service, which had a detrimental effect and led to overwhelming feelings of discouragement:

*“Well of course there’s waiting lists, that’s a big issue. You know, sometimes people are waiting four to six months to get in somewhere.” (Participant 01; 63-year-old woman)*

Participants explained that wait lists were a particular issue due to the fleeting nature of their motivation, and the subsequently time-sensitive window of opportunity to seek and accept support. As such, participants described the importance of being able to receive support immediately once they were ready and willing to seek it:

*“Especially for getting into treatment centres and stuff, and when you need help right away. I feel like people are dying out there … people just need to get into a place where they can detox. They just need that date. I’ve known many people that have died … I OD’d before I went to treatment this time. You just never know, that week could make a difference.” (Participant 36; 33-year-old woman)*

Further, participants expressed a need for an increase in hours of operation since many of the services they rely on have limited hours, rendering them non-conducive to participant’s lifestyles and schedules:

*“[The harm reduction service] closes very early, I don’t like the hours there. If they could do evening hours, that would be great. That’s important. Yeah even from 6 to 8, or 9 even.” (Participant 24; 55-year-old woman)*

Additionally, participants suggested that certain services (i.e., private for-profit counseling or residential treatment centres) were altogether unaffordable, which hindered their ability to reach their substance use goals:

*“I wish I found like a rehab program that you didn’t have to pay 30 grand for. I was looking around for something like that before I got into the trouble and I didn’t have much luck, they were all pay-for.” (Participant 21; 28-year-old man)*

#### Scale-up specific services and related supports

While the majority of participants felt supported by the services offered in their community, many indicated an overall lack of community-based services, as well as a lack of specific services tailored to meet their particular needs. This was particularly true for smaller or more rural communities. For example, one participant explained:

*“In our community, there’s no detox, there’s no safe use site, no safe injection site, no rehab, there’s no inpatient treatment centre … there’s nowhere to go.” (Participant 02; 43-year-old woman)*

Participants indicated that they either had to travel outside their community for certain services, or simply did not receive support. While a general lack of services was common for many participants and communities, there were concrete suggestions put forth by participants to scale-up harm reduction services, counseling, treatment, and housing.

##### Harm reduction services

Many participants expressed a need for their communities to implement more harm reduction services. Specific suggestions included both temporary and permanent overdose prevention/supervised consumption services (with multiple booths that allow consumption of any substance via any route of administration), as well as needle exchange services and naloxone distribution programs:

*“If there were even temporary safe injection sites at those locations in the city [with the biggest concentrations of overdoses], they would probably be better accessed and an immediate resource or help for people who are overdosing in that moment … Also, these Naloxone kits, I would be one to advocate that merchants in the downtown core all have access to those.” (Participant 04; 44-year-old man)*

In particular, participants expressed that anonymous mobile needle distribution/outreach services were helpful because they provided safe supplies, collected used ones, and offered a range of additional health and social services which obviated many of the physical (e.g., accessibility) and psychological (e.g., stigma) barriers participants commonly encountered when accessing services:

*“Needle exchange is really good because you can just call them you don’t have to give them your name, nothing, and just tell them where you are, anywhere, and they’ll come meet you … It’s mobile … They give you literally everything to keep it clean and safe, right? Because they don’t want people sharing needles, right? And so, it’s actually one really good thing.” (Participant 13; 35-year-old man)*

Participants described the importance of anonymity and accessibility, and as such, suggested a need for low-barrier community and addiction-related drop-in centres that do not have burdensome application (e.g., paperwork) or administrative (e.g., identification) requirements. Participants recommended that these drop-in centers could assist people with a wide range of needs, and would be accessible due to being located in one centralized building or location. For instance, when asked about what services were needed in their community, one participant described:

*“Drop-ins - where people can use the computers, get counseling, housing, the help you need.” (Participant 24; 55-year-old woman)*

Another participant described a similar desire for access to a low-barrier comprehensive service design in their community, as based on their previous experience utilizing such a service when they lived in a larger city:

*“There’s nothing else here. When I was in [city name] I was at the community centre a lot right?...There’s the safe injection site there, but then there’s also counselors, and doctors, and everybody there that you can talk to. It’s open from 8 in the morning to 8 at night … like a drop-in centre.” (Participant 08; 32-year-old man)*

While participants provided examples of suggested supports that could be offered within low-barrier centralized services, emphasis was placed on the need for connections between detox and short- and long-term residential treatment that were free of cost:

*“I think [service name] in [city name] offers medical detox in-house, and that has had a really good impact. You’re medically detoxed there, and then you go right into a 30 day program. But the problem is that it’s like 30 thousand dollars.” (Participant 11; 37-year-old man)*

Participants further indicated that harm reduction services are a great place to initiate substance use support, particularly as they consider each person’s unique substance use goals when offering services:

*“For someone who is starting, the idea of harm reduction is a good starting point … not only is relapse common, but it’s likely.” (Participant 33; 57-year-old man)*

However, some participants indicated that they were personally not able to use substances in less harmful ways, and that their substance use was all-or-nothing, and they therefore required abstinence-based recovery supports and services:

*“Yeah, so when I started to try and get clean I was doing the outpatient group … but it’s a harm reduction [based program], so it’s not necessarily like an abstinent program. After going to treatment I’m learning a lot more about addiction, like that that won’t work for me. I needed to stop.” (Participant 38; 30-year-old woman)*

##### Counseling

In addition to harm reduction services, participants indicated a need to scale-up counseling services in their communities. Nearly all participants had experiences with counseling, including one-on-one and/or group formats, and many had experiences with both. The settings in which participants received counseling varied and was quite broad; participants reported receiving counseling from different types of professionals (psychiatrists/psychologists, counselors, therapists, addiction or social workers, peer-support specialists, etc.) and in a variety of locations (through low-barrier drop-in clinics, hospitals, mental health and addiction clinics, residential or day treatment, private practice, etc.). Most participants preferred one-on-one over group counselling and did not feel comfortable opening up and expressing themselves in group settings. Moreover, many found that group settings could be a trigger for substance use relapse, especially when people in the group were not at the same stage of recovery or did not have the same substance use goals:

*“When I go to my support group, there’s lots of drug users that are active and they’re asking and trying to get us to use. So it’s not really helpful because they’re supposed to be encouraging the recovery, not the active use.” (Participant 05; 30-year-old woman)*

Participants further explained that trauma-informed counseling was the most beneficial and needed as it emphasized the role of traumatic experiences in shaping peoples’ substance use trajectories, and enabled participants to address the underlying issues that they felt had contributed to their substance use:

*“[Counseling] is really helpful if you’ve got trauma from when you were a child. Like I’ve got trauma from when I was a child. They’re helping me to overcome that and deal with the problem. My counselor tells me if I keep that inside and don’t talk about it, it builds up and you’re gonna wanna use. For me, I find talking about it, I don’t want to use again.” (Participant 25; 26-year-old man)*

Additionally, specific counselor characteristics were highly regarded as important determinants of participants’ motivation and willingness to engage in counseling sessions. Specifically, counselors who were caring, empathetic, non-judgmental and/or had their own lived experience with substance use (i.e. PWUD) were preferred and requested. PWUD counselors were often perceived as approachable, valued, and trustworthy counselors, mainly in comparison to counselors who were taught ‘by the book’ and did not have lived experience with addiction. Some participants therefore indicated that PWUD are best suited to assist clients due to their personal experience and ability to understand the client’s needs:

*“My counselor used to be an addict 26 years ago, so she knows everything and she’s already been through what I’ve been through, and I find it’s very useful to me because I’m talking to somebody who’s already been through the exact same thing that I’ve been through.” (Participant 25; 26-year-old man)*

Specific counseling formats suggested by participants included one-on-one as well as daily drop-in groups, as well as familial or grief counseling. Notably, in line with the harm reduction model, participants were clear that counselors (regardless of format or credentials), needed to actively listen to their needs and develop a functioning therapeutic relationship where they could work together towards reaching the participant’s unique substance use goals.

##### Treatment

Many participants indicated a need for more long-term rehabilitation/residential inpatient treatment programs since they provided the stability required to meet their substance use goals. For example, when asked which service utilized was the most beneficial, one participant indicated:

*“Probably actually going to rehab itself. Well there was 19 days, or almost 3 weeks of a safe environment where you’re not gonna use. You don’t have to worry about work or stress. That set me up for the best success.” (Participant 11; 37-year-old man)*

Participants expressed the importance and need for affordable (ideally free) long-term residential treatment programs that did not have pre-determined end-dates, which would provide participants with adequate time to heal, while providing the foundation needed to reach their substance use and other goals:

*“There needs to be more money put into funding places that doesn’t [sic] have an end date right? You’re there to heal yourself. [Healing] doesn’t happen in 90 days, 60 days, 30 days. It’s something that you continue to work on. If the government could realize that and give a little bit more funding, you’re gonna fix a lot of addiction.” (Participant 32; 31-year-old man)*

However, some participants expressed the benefits of aftercare/drop-in outpatient programs, and indicated that upon completion of a residential treatment program, they relied on these services to maintain sobriety and/or meet their substance use goals. Therefore, residential treatment was largely conceptualized as a requirement needed to build a strong foundation and to be able to be connected with outpatient care:

*“The program I’m in now is [helpful] because I was able to go to a treatment centre first … after the treatment centre I was able to get right into this program, and it’s gonna be able to give me a foundation.” (Participant 36; 33-year-old woman)*

Therefore, it was often the combination of both of these types of treatment that many participants suggested was beneficial and needed.

##### Housing

Lastly, additional housing support, including both temporary housing/shelters and longer-term transitional housing such as recovery houses and/or sober living environments, were suggested by many participants:

*“A lot of people are going into sober living houses up in [city name] … we don’t have that … supportive living environments would absolutely be really beneficial.” (Participant 04; 44-year-old man)*

Specifically, participants articulated a desire for housing at two distinct and crucial time points: 1) during the waiting period between detox and residential treatment, and 2) after the completion of residential treatment. To illustrate, one participant suggested:

*“You know the detox will only house you for a few days and there’s one spot for safe beds. So maybe a dedicated place that has safe beds for people that want to get into a treatment centre, and they’ll keep you until your date where you leave to go to treatment. That would probably be really good.” (Participant 41; 37-year-old man)*

In terms of the provision of post-treatment housing, many participants indicated the need for affordable recovery houses, and expressed that this was vital to ensuring that the healing process and ‘foundation’ built through treatment was not futile. For instance, when asked what services or supports they needed in their community, one participant explained:

*“Recovery houses, transitional housing. Once people get out of treatment and they’re coming back to the city because that’s where they’re from, they’re right back in the same environment that they got out of. It’s just the same cycle again. We need to see more of those.” (Participant 04; 44-year-old man)*

Participants also expressed the importance of providing transitional housing in terms of cost-effectiveness, and highlighted the futility of paying to send someone to treatment, and then losing that financial investment by not providing them with a safe place to live and maintain their goals afterwards.

## Discussion

This study qualitatively examined substance use service needs among a sample of PWUD with treatment and service experience in Ontario, Canada. The results were informed by expert end users’ experiences and provide insight towards expanding the evidence base for appropriate substance use policy and program planning and closing the treatment gap in the province.

Study results highlighted the detrimental effect stigmatization can have on service access among PWUD, as many study participants indicated that they have experienced judgmental services and staff when seeking support, and that this deterred them from following through with treatment plans or goals. As such, our study results correspond with prior literature demonstrating how stigmatization directly influences and aggravates existent service accessibility issues among PWUD [57–61]. For instance, a US-based qualitative study found that many PWUD experience both structural and individual stigmatization, which manifests in overt discrimination when accessing health care services and deters them from participating in evidence-based treatments [57]. Study participants suggested ways to decrease stigmatization, including amending and enhancing the professional educational system to be more client-based and trauma-informed to help reduce stigmatic professional practice and work culture, as well as employing and utilizing PWUD as peer counselors and service facilitators to ensure that services are provided in a non-stigmatizing and relatable manner. Employing PWUD within services and drawing on their expertise is becoming increasingly common, both internationally and in Canada specifically, and has been shown to result in better health outcomes compared to treatment-as-usual as it can provide a more approachable point of contact and increase treatment and service uptake [62–68]. For instance, peer-delivered services have been associated with positive impacts on levels of hope, empowerment, and quality of life [69]. This is important since enhancing empowerment among PWUD has been identified as a key process in substance use recovery since it can enfranchise both the peer worker and client to overcome self-shaming thoughts and attitudes, and increase self-efficacy [68].

While incorporating peer support into service frameworks has been increasingly advocated for, there is a lack of consensus around what constitutes peer practice, as well as the specific qualifications needed (including ongoing debates regarding whether PWUD must be in active recovery/sober in order to be qualified), which can vary by role and organization [50, 70, 71]. Moreover, while peer involvement can enhance feelings of comfort among PWUD seeking services, many peer workers report issues around workplace equity, tokenism, power and pay inequities including receiving minimal financial compensation, coupled with considerable job burnout [71, 72]. Furthermore, a review of peer-identified barriers to successful implementation of peer roles suggested that many peer workers experience lack of credibility, negative attitudes from other professionals, and struggle to find their place and identity within their roles [73]. These issues suggest the need for stronger and clearer organizational policies and guiding principles that take peer perspectives and experiences into consideration, and outline best practices for integrating peer workers into existent service designs.

Additionally, our results underscore a systemic gap in service delivery and a severe lack of coordination between various substance use services available for PWUD in Ontario; these commonly resulted in relinquished efforts to seek support, and continued or escalated substance use among participants. However, these issues are not novel [21, 74–76]. Literature has highlighted that PWUD face numerous barriers at the patient (e.g., motivation, stigma), program (e.g., wait times) and system (e.g., lack of coordination) levels while trying to navigate services [74, 77]. A lack of inter-program coordination and collaboration, and how this deters treatment completion and successful transitions, has specifically been noted within the Ontario context [78]. This lack of coordination contributes to the ‘revolving door’ nature of substance use service systems, where clients often do not receive timely and adequate support and/or end up frequenting services in a cyclical manner. It is therefore imperative that participants are adequately connected and retained in services in order to reduce unnecessary treatment readmissions.

System fragmentation and lapses between detoxification and residential treatment services were particularly emphasized by participants, and are a longstanding issue within substance use treatment models. These gaps are commonly due to capacity issues and mismatches between referrals and clients’ needs, which often leave PWUD on lengthy wait-lists and vulnerable to relapse [42]. Thus, some of the highest treatment completion rates have been reported when detoxification and residential treatment services are fully integrated (i.e., contained in the same setting), and such single-setting systems have been found to provide seamless transitions between all levels of substance use care [77, 79–81]. This is consistent with suggestions from many study participants for an integrated, cohesive service and referral model that would help close treatment gaps and ensure participants no longer ‘fell through the cracks’ of the system.

Low-barrier drop-in programs such as rapid access addiction medicine (RAAM) clinics (i.e., harm reduction-based walk-in clinics which provide PWUD with immediate access to a range of pharmacotherapeutic and psychosocial addiction services without a referral) are one such service model which have been improving transitions between providers [42, 82]. These low-barrier service designs can provide the anonymity and accessibility participants require, can strengthen coordination between defined care pathways, and can be used as a starting point to seamlessly connect and refer PWUD to wrap-around services and individualized treatments that address their unique needs [82–84]. Preliminary results examining the effectiveness and feasibility of such comprehensive service models highlight their ability to improve health outcomes by combining substance use care and harm reduction with access to primary care, as well as social and psychological support including counseling and housing (two key needs expressed by study participants) [83, 84]. Additional low-barrier programs that should be incorporated into an integrated model of substance use care are outreach services and technological (i.e., mobile applications) and/or telehealth-based programs that can reach underserved PWUD, including those who reside in remote/rural areas with limited services. These programs have the potential to reduce disparities in the delivery of care, and have become increasingly utilized and integrated into the provision of substance use treatment for PWUD [85, 86]. Extant literature highlights that mobile/telehealth is an effective low-barrier option for treatment that can result in high completion rates and client satisfaction, and has been found to decrease risky substance use practices (e.g., injection drug use) [85–87].

Since PWUD have complex, individual, needs and require different types of care, it is important that they have access to a range of low-barrier support options, and that any treatment model is client-oriented and allows PWUD to have a voice in their care and to set their own goals and treatment plans, co-developed with supportive care providers. Notably, the importance of integrated models of substance use service delivery as suggested by participants is becoming increasingly recognized, and uptake and implementation of these models of care is slowly increasing [83, 84]. For example, the Ontario provincial government recently established a Mental Health and Addictions Centre of Excellence (as well as Ontario Health Teams to help with regional coordination), which will oversee the delivery and quality of substance use service provision with the aim of standardizing care, expanding service and access, reducing wait times, and helping clients navigate the system [88–90]. The strategy is committed to employing a collaborative approach to expanding harm reduction services and supports to meet clients’ needs throughout their recovery journey. This is vital since the absence of PWUD voices and involvement in program design and delivery has resulted in ineffective service provision thus far. With this in mind, calls have been made to the government to ensure that the new model offers better access to innovative cohesive programs such as RAAM clinics/centralized referral programs, and mobile/telehealth options that increase access to the continuum of care and offer more individualized case management [91].

Whether the substance use service system in Ontario can be overhauled to truly address PWUD’ multifaceted treatment needs remains to be seen. Regardless, in order to address the unmet treatment needs of PWUD in Ontario, it is crucial that governments, frontline substance use service organizations, and PWUD work together to establish a course of action that ensures PWUD have a range of low-barrier treatment options and are meaningfully integrated into service provision and effectively supported.

### Strengths and limitations

The strength of the current study includes a diverse sample of PWUD participants from a variety of communities, backgrounds, and experiences, enabling us to assess a wide range of substance use service needs for PWUD in Ontario. However, our sample size was small and based on convenience sampling via substance use services, rendering our results non-generalizable; results should be considered a snapshot of current needs among a select sample of treatment-seeking English speaking PWUD located in Ontario. Moreover, face-to-face interviews were conducted primarily within residential treatment settings, which may have influenced or unconsciously biased these participants’ responses.

Future research examining service and treatment needs of PWUD should attempt to examine and evaluate the feasibility and effectiveness of comprehensive integrated service models, as well as include larger, more representative samples.

## Conclusion

Substance use service provision in Ontario is severely fragmented, resulting in gaps in treatment for PWUD that remain despite government efforts to address this issue. As Canada’s opioid overdose crisis persists, it is increasingly necessary that the needs of PWUD – as experts and end users of services – are taken into consideration and valued. Notably, this study highlighted recommendations and areas for policy change and program delivery for substance use care in Ontario. Suggestions include the implementation and scale-up of a government-funded, low-barrier, integrated and cohesive model of substance use service provision and referral that works in meaningful collaboration and employs PWUD to address their unique experiences and needs.

## Supplementary Information

**Additional file 1.**

## Data Availability

A de-identified dataset used and/or analyzed during the current study are available from the corresponding author on reasonable request.
