# Supplementary material for: A qualitative examination of substance use service needs among people who use drugs (PWUD) with treatment and service experience in Ontario, Canada
Source: BMC Public Health. 2021 Nov 6;21:2021. doi: 10.1186/s12889-021-12104-w (PMC8571863; doi:10.1186/s12889-021-12104-w)
Supplement: Supplementary file 1 — Additional file 1. [file 12889_2021_12104_MOESM1_ESM.docx]

**Supplemental Material: Semi-Structured Participant Interview Guide**

1. Are there any service(s) in your community that you use (or have used) for your drug use?

- If yes: how did you first learn about this (or these) service(s)?
- If yes and more than one service: Which service is the most helpful, and why? Which is the least helpful, and why?
- If no: Can you tell me more about this? Are there services available but you cho(o)se not to use them? If so, how come?

1. As a person who identifies as having a lived experience with drug use, do you feel you have access to enough resources, supports and/or services to be healthy and safe in your community?
   - If yes: can you tell me more about this? Is there anything in specific that contributes to this?
   - If no: can you tell me more about this? Why not?
2. Are there any resources, supports, and/or services that you feel are needed or you would like to see for people who use drugs in your community that are not currently available or accessible?
   - If yes: can you tell me more about these? How would these be helpful? Why are they needed?
   - If no: can you tell me more about this? Why not?
3. Are there any barriers or factors that make it difficult to access services in your community?
   - If yes: can you tell me more about these barriers? What could be done to reduce or eliminate them?
   - If no: can you tell me more about this? Why not?

Typically, people who have lived experience with drug use have been excluded from research related to drug use, but have extremely valuable input as end-users of the services. With this in mind, as a person who identifies as having lived experience with drug use, I am going to ask you a couple questions about research related to drug use.

1. Are there any specific topics related to drug use that you think would require further research?
   - (Example prompts: specific drugs? Interventions for drug use?)
   - If yes: can you tell me more about this? Is there a reason that you feel it requires further research?
   - If no: can you tell me more about this? Do you feel that there is enough research?
2. Are there particular sub-populations or groups of people that you think drug use research should focus on?
   - (Example prompts: youth, new users, Indigenous, LGBTQ2S+, homeless, etc.)?
   - If yes: can you tell me more about this? Is there a reason that you feel these sub-populations should be a focus for drug-use research?
   - If no: can you tell me more about this? How come?
3. Do you think that people who use drugs should be involved in research?
   - If yes: How so? Can you tell me more about this?
   - If no: How come?
4. It is important that research on drug use gets to the people who can benefit from the information the most, such as people with lived or living experience with drug use. What do you feel is the best way to inform people or let them know about important research findings that could be relevant to them or they may want to know?
